# A membrane permeability database for nonpeptidic macrocycles

**DOI:** 10.1038/s41597-024-04302-z

**Published:** 2025-01-03

**Authors:** Qiushi Feng, Danjo De Chavez, Jan Kihlberg, Vasanthanathan Poongavanam

**Affiliations:** Department of Chemistry-BMC, Uppsala University, SE-75123 Uppsala, Sweden

**Keywords:** Cheminformatics, Databases

## Abstract

The process of developing new drugs is arduous and costly, particularly for targets classified as “difficult-to-drug.” Macrocycles show a particular ability to modulate difficult-to-drug targets, including protein-protein interactions, while still allowing oral administration. However, the determination of membrane permeability, critical for reaching intracellular targets and for oral bioavailability, is laborious and expensive. In silico methods are a cost-effective alternative, enabling predictions prior to compound synthesis. Here, we present a comprehensive online database (https://swemacrocycledb.com/), housing 5638 membrane permeability datapoints for 4216 nonpeptidic macrocycles, curated from the literature, patents, and bioactivity repositories. In addition, we present a new descriptor, the “amide ratio” (AR), that quantifies the peptidic nature of macrocyclic compounds, enabling the classification of peptidic, semipeptidic, and nonpeptidic macrocycles. Overall, this resource fills a gap among existing databases, offering valuable insights into the membrane permeability of nonpeptidic and semipeptidic macrocycles, and facilitating predictions for drug discovery projects.

## Background & Summary

Developing a new drug from discovery to market is an expensive and time-consuming process^1^. Approximately half of the targets associated with human diseases are classified as “difficult-to-drug” with traditional molecules following Lipinski’s Rule of 5 (Ro5)^2^, which outlines limits for molecular weight (MW ≤ 500 Da), calculated lipophilicity (cLogP ≤ 5), as well as hydrogen bond donors and acceptors (HBD ≤ 5, HBA ≤ 10). Although biologics may be suitable for difficult-to-drug targets, their lack of cell permeability hinders access to intracellular targets and renders them unsuitable for oral administration. Recent research has shed light on the opportunities provided by compounds that reside outside the Ro5 boundaries, i.e. in the beyond Rule of 5 (bRo5) chemical space^3–5^. Among these compounds, macrocycles, characterized by a ring of at least 12 atoms, exhibit the capability to modulate difficult-to-drug targets, including those with tunnel, flat, or groove-shaped binding sites, as well as protein-protein interactions (PPIs), while still allowing for oral administration^5–7^.

Independent of chemical space, solubility, cell permeability and a not too high metabolism in the liver are the three most important determinants of the oral bioavailability of drugs. Optimizing this triad of drug properties becomes increasingly difficult as compounds grow in size, putting macrocycles and other compounds in the bRo5 space at higher risk. Despite the recent emergence of macrocyclic peptides as a promising chemical class in drug discovery^8–10^, they often suffer from issues with solubility, cell permeability and metabolic instability^11^. This originates from the high polarity of amide bonds in the peptide backbone^12^, and any polar groups in their side chains. In contrast, nonpeptidic macrocycles do not carry the burden of a polar backbone and more often display both cell permeability and oral bioavailability^6^.

Measurement of cell membrane permeability of drugs is not only crucial to assess their ability to reaching intracellular targets, regardless of their location in the central nervous system (CNS) or peripheral sites, but is also utilized as a model system for estimating oral absorption^13^. Various *in vitro* assays are employed to measure cell permeability, including the human colorectal adenocarcinoma cell line (Caco-2), Madin–Darby canine kidney (MDCK) cells, and the low-efflux MDCK clone Ralph Russ canine kidney (RRCK). The parallel artificial membrane permeability assay (PAMPA) provides a cost-effective assessment of passive membrane permeability in a cell free system, while the cell-based assays provide data that is more relevant for permeability and oral bioavailability in an *in vivo* setting. However, generating experimental permeability data is both time-consuming and expensive, in particular in cell-based systems. Alternatively, *in silico* methods are not only cost-effective but also sufficiently accurate and fast enough to be used as high-throughput filter in the drug discovery projects, enabling predictions before compound synthesis and testing^14^.

To facilitate the development of accurate and efficient computational predictions, it is crucial to collect and curate experimental data with structural information, making it available to scientific communities as per the FAIR guideline (Findable, Accessible, Interoperable, and Reusable)^15^. In this study, we report the construction of a membrane permeability database for 4216 macrocycles, ranging from nonpeptidic to semipeptidic, which has been collected and curated from the scientific literature, patents, and various bioactivity data repositories. This comprehensive online resource comprises structures annotated with molecular descriptors and permeability data obtained from different assays and endpoints. It is readily accessible and downloadable through the webserver (https://swemacrocycledb.com/). Our database is complementary to the CycPeptMPDB^16^, a comprehensive database of membrane permeability for more than 7000 cyclic peptides.

## Methods

### Data collection and curation

**Fig. 1:**
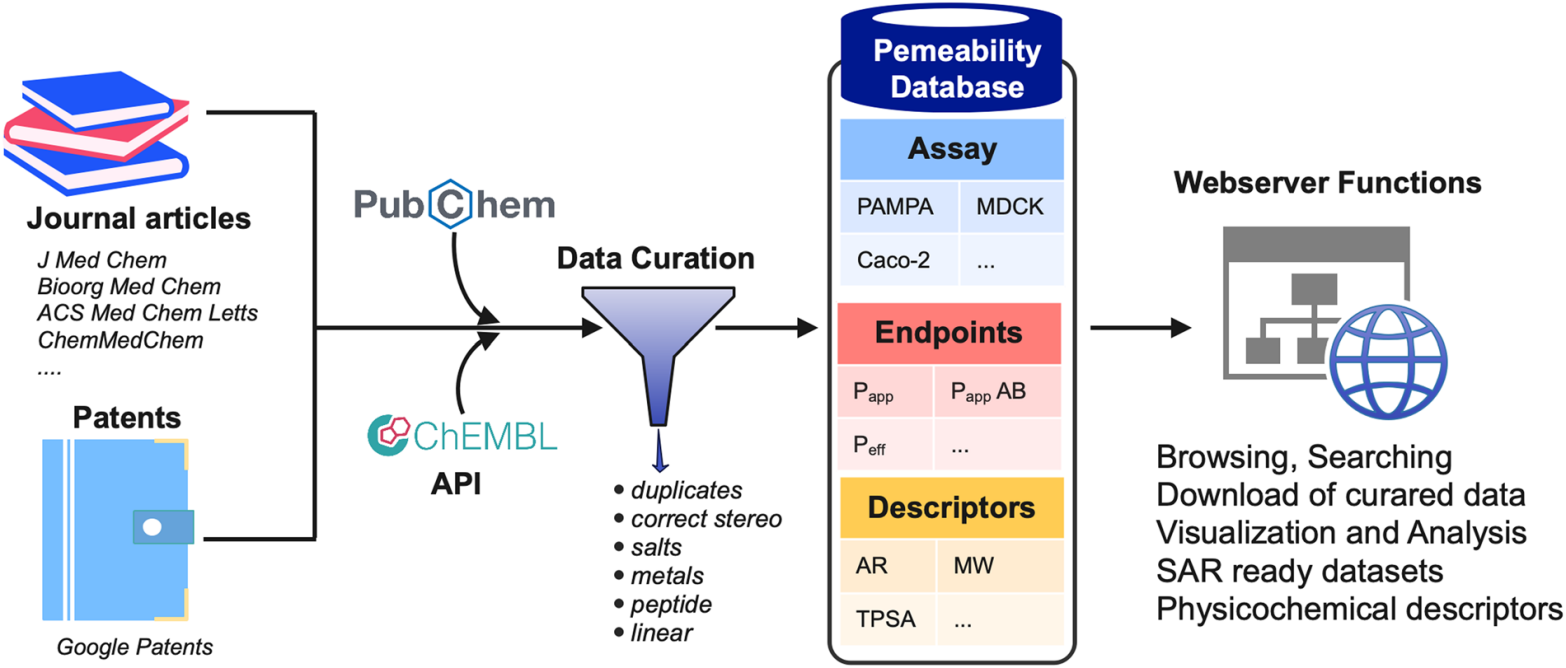
The workflow for construction of the membrane permeability database for nonpeptidic macrocycles and its functions. Structures and permeability data were retrieved from the literature, patents, and scientific databases, followed by manual curation. The webserver provides readily downloadable datasets for macrocycles evaluated in various membrane permeability assays often with different endpoints, as well as the structures and molecular descriptors of the macrocycles.

Macrocycles exhibiting membrane permeability were gathered from three different sources: 1) the scientific literature, 2) patents, and 3) public repositories and then incorporated in the database (Fig. 1, Supplementary Table 1). PubMed^17^ and Google Scholar were used to search the literature and identify macrocycles for which permeability data has been published. Keywords like “macrocycle” were combined with either the general term “permeability” or specific assay names (Caco-2, PAMPA, MDCK, RRCK) to query scientific journals. A similar search was done in Google Patents to collect approved patents which disclosed nonpeptidic macrocycle membrane permeability data. In a further attempt to gather data comprehensively, the ChEMBL database^18^ was mined using its Python web resource client; the query python code can be found in GitHub. The RDKit Molecule Substructure module^19^ was employed to filter macrocycles, defined as having a ring with at least 12 heavy atoms. Subsequently, all structures (SMILES) and cell permeability data were imported into Molecular Operating Environment (version 2022.02)^20^.

The dataset underwent manual curation, involving the removal of mixtures, inorganics, salts, solvent molecules, and also structural normalization. Descriptors for polarity (HBA and HBD) are highly influenced by the protonation state of the molecules^6^. Since predictions by different tools often yields different charge states for the same molecule^21^, we treated molecules as ‘uncharged’ for calculation of their descriptors. To allow analysis and model building permeability values were standardized, first by conversion to the unit cm/s and then by calculation of their logarithmic values. For permeability values reported with a “>” or “<” sign, this was retained in both the original and standardised values. There are 36 compounds having such undefined values in the dataset. Overall, the collection and curation resulted in a database containing 4216 diverse and unique macrocycles and 5638 permeability datapoints. In the future, the database will be updated on a biannual basis.

### Quantification of the peptidic nature of macrocycles

No standardized and quantitative definition exists for the peptidic nature of macrocycles. We propose that the amide ratio (AR, Eq. 1) is a relevant and intuitive descriptor of the peptidic nature of macrocycles. Calculation of the AR is based on the number of amide bonds (nAB), including both NH and N-alkylated ones, within the macrocyclic ring, multiplied by three to account for the number atoms (**-C-N**-**C**_**α**_-) forming each amide bond. Division by the macrocycle ring size (MRS), i.e. the total number of atoms in the macrocyclic ring, then provides the AR.

1 $${\rm{AR}}=({\rm{nAB}}\times 3)/{\rm{MRS}}$$

AR returns values between 0 and 1, a value of 0 represents a completely nonpeptidic macrocycle, and 1 represents a full cyclic peptide. We also propose that macrocycles having an AR of from 0 to 0.3 are classified as nonpeptidic, those with an AR between 0.3 and 0.7 as semipeptidic, while an AR > 0.7 characterizes macrocycles which are mainly peptidic. The AR is identical to the recently reported the ‘Peptide Character Index’^22^. However, we have proposed thresholds to distinguish between nonpeptidic, semipeptidic, and peptidic macrocycles, which have been thoroughly validated using known datasets (cf. Quantification of peptide and nonpeptide macrocycles, below). Additionally, the code for calculating the AR metric is freely available.

### Webserver implementation

The webserver implemented in this study was built on the Django web framework (version 3.2.23). The development of the web interface involved the use of standard web technologies, including HTML5, CSS, and JavaScript, with all data within the web server stored and managed using SQLite, a lightweight and efficient relational database management system. RDKit (version 2023.9.5)^19^ was employed for molecule visualization. Specifically, RDKit was used to generate structures that include the stereochemistry of the macrocycles^23^, and convert the resulting isomeric SMILES into PNG and SDF files within the web interface. ECharts (version v5.5.0) was utilized to support online data visualization^24^. The functionality for table sorting and filtering was implemented using DataTables (https://www.datatables.net/), a JavaScript library for enhancing HTML tables. The website has been thoroughly tested to ensure functionality across multiple operating systems and web browsers. Most of the codes used in this work are open-source and properly acknowledged. The code for the final version of the web server is provided on GitHub.

## Data Records

**Fig. 2:**
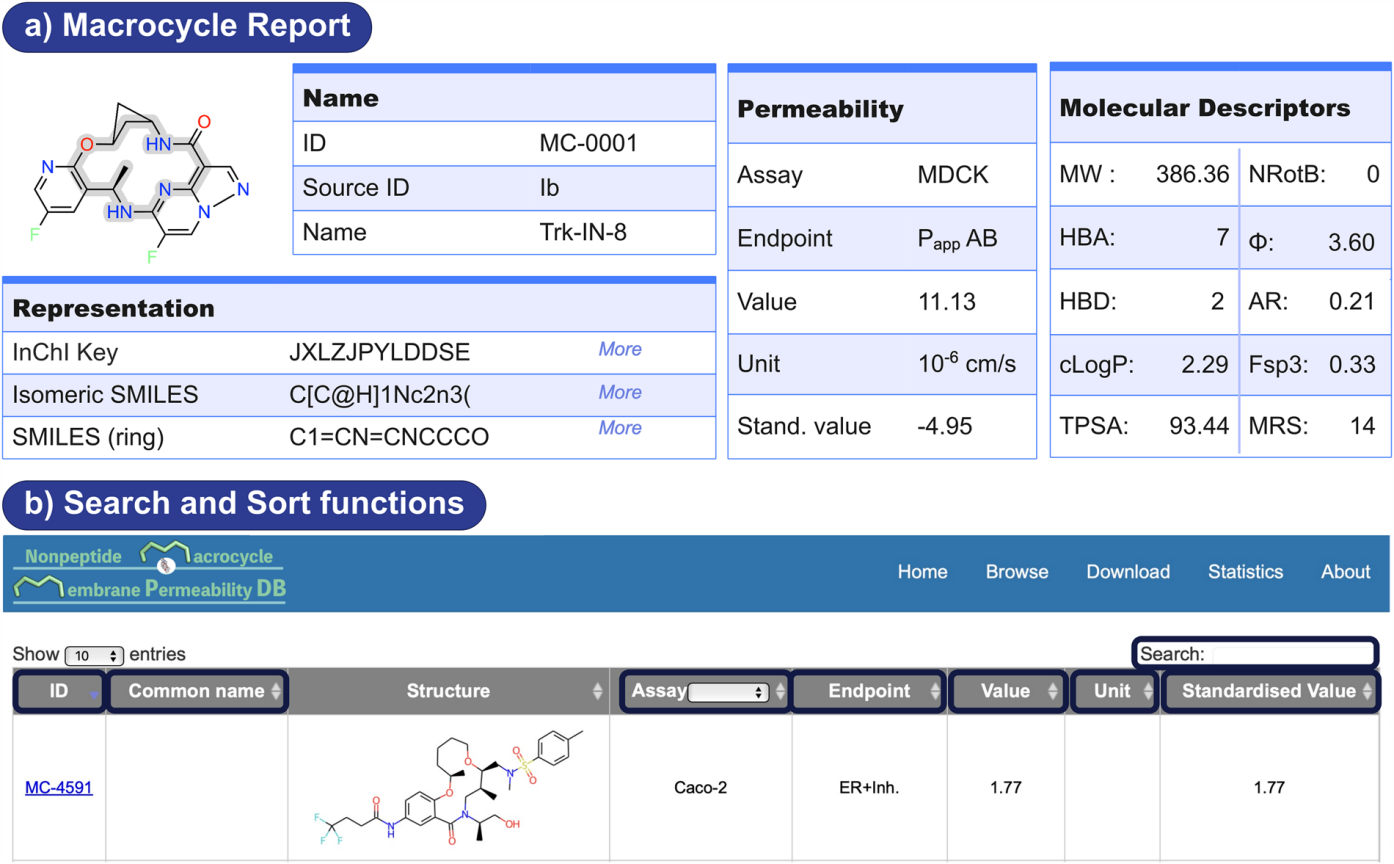
Schematic representations of (**a**) a report which is available for each permeability entry in the database and (**b**) the ‘Search’ and ‘Sort’ functions. *Abbreviations*: MW: Molecular weight; HBA: Hydrogen bond acceptor; HBD: Hydrogen bond donor; cLogP: Calculated lipophilicity; TPSA: Topological polar surface area; NRotB: Number of rotatable bonds; Φ: Kier flexibility Index; AR: amide ratio; Fsp3: fraction of sp3 carbon atoms; MRS: macrocyclic ring size.

The structures of the 4216 unique macrocycles, their molecular descriptors and the 5638 permeability datapoints reported for them are available on the https://swemacrocycledb.com/ web server (Fig. 2a). A unique molecule ID identifies each macrocycle, for which multiple permeability measurements may have been reported and included in the database. Three categories of information is provided for each permeability measurement: (i) a *Representation*, where the structure, InChI Key, isomeric SMILES for the overall macrocycle and the SMILES for the macrocyclic ring is shown; (ii) *Permeability* information containing the type of permeability assay, the endpoint, value, and unit; and (iii) key *Molecular Descriptors* for the macrocycle, including the descriptors of Lipinski´s^2^ and Veber´s^25^ rules, as well as other descriptors of macrocycle flexibility and structure.

Due to the high computational cost and uncertainty in the conformational sampling of macrocycles^26,27^, this database does not provide conformations. Instead, we provide isomeric SMILES (contains chirality information) and descriptors for each macrocycle, as described above. The original sources from which the structure and permeability data were extracted are also available for the user. In order to provide ready-to-use datasets for QSAR modelling, all membrane permeability values were standardized into logarithmic values. All data records incorporated in the database are ready to download. The browse menu also offers multiple search and sorting options, primarily by unique ID, common name, permeability assay, endpoint, permeability value, unit and standardized permeability value (Fig. 2b).

In addition to the web server resource (https://swemacrocycledb.com/), the peer-reviewed version 1.0 of this database has been archived as a static repository on Figshare (10.6084/m9.figshare.26964259)^28^. The repository is organized into two main directories, “Data” and “Code,” with a README file to guide users through the directory structure and contents. The Data directory is divided into three subdirectories, each containing membrane permeability data for specific endpoints in comma-separated values (.csv) format. A 2D representation of each compound is also available as an image file (.png) and in structured data file (.sdf) format, identified by a unique macrocyclic ID for consistency and easy reference. The Code directory includes a Jupyter Notebook documenting the step-by-step processing and data analysis workflow. This notebook allows users to directly access and run the code used for data extraction and preprocessing of macrocycles with membrane permeability data, enhancing reproducibility.

## Technical Validation

### Membrane permeability database statistics

#### Sources

**Fig. 3:**
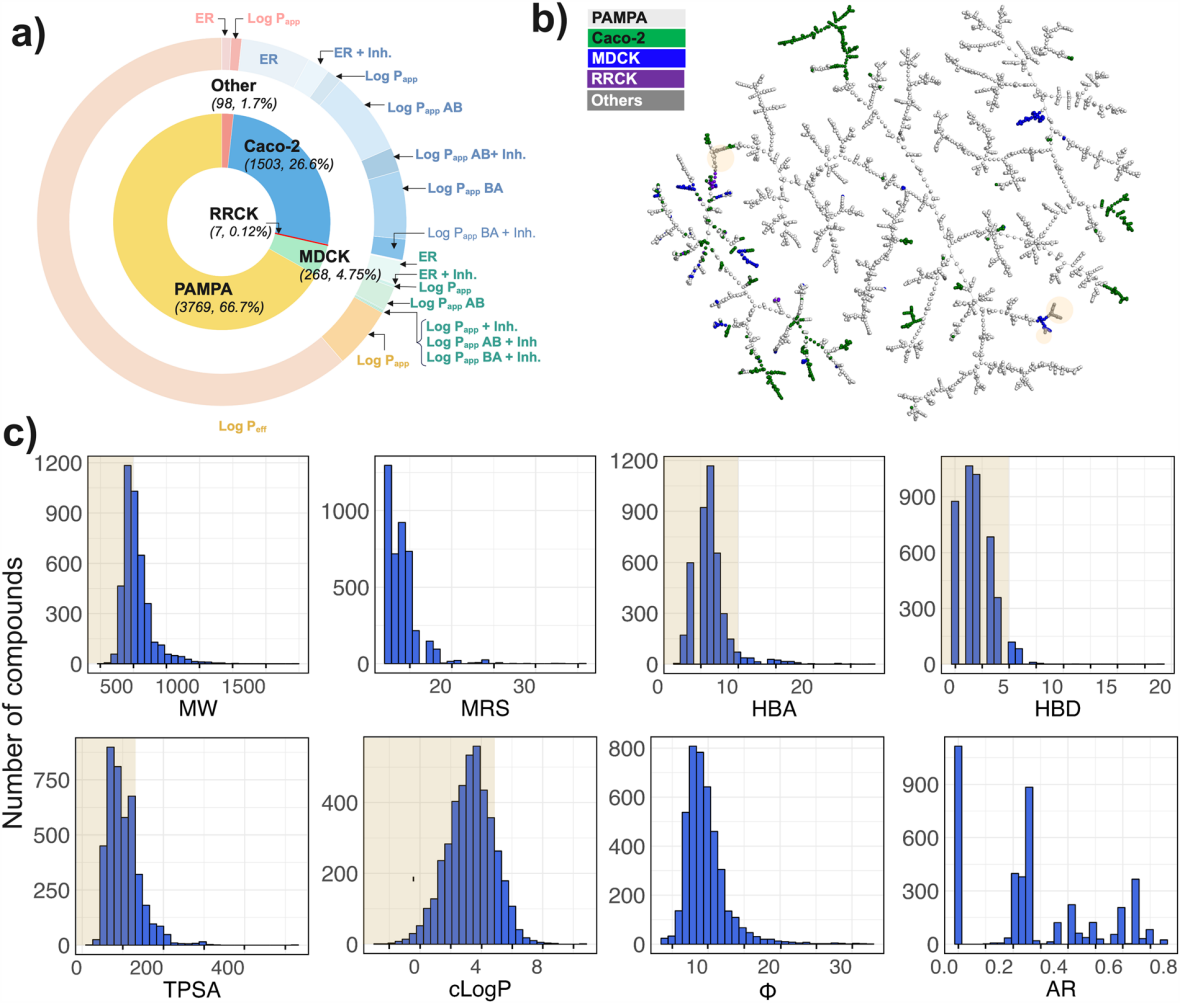
(**a**) Nested-pie chart of the permeability data for the macrocycles in the database. The different types of permeability assays are indicated in the inner ring, endpoints in the outer ring. (**b**) TMAP visualization of the structural diversity of the nonpeptidic macrocyclic dataset (*n* = 4216). The different types of permeability assays are highlighted on the tree. (**c**) Molecular property distribution of the macrocycles included in the database, as described by 2D molecular descriptors representing size, polarity, a lipophilicity, flexibility, and amide ratio. The upper limits of the descriptors of the Ro5 and Veber´s rule are indicated by grey shading. *Abbreviations*: MW: Molecular weight; MRS: macrocyclic ring size; HBA: Hydrogen bond acceptor; HBD: Hydrogen bond donor; TPSA: Topological polar surface area; cLogP: Calculated lipophilicity; Φ: Kier flexibility Index; AR: amide ratio.

The dataset reported herein consists of 5638 permeability datapoints for 4612 macrocycles, collected from 103 scientific articles and 9 patents published during 2006–2023 (*last updated July 2023*) as well as data from the ChEMBL database. Out of the 5638 datapoint records, 84%, 4%, and 11% are from scientific articles, patents, and the ChEMBL repository, respectively. ***Assays:*** The dataset has been divided into five membrane permeability assay categories (Fig. 3a and Table 1), namely, PAMPA, Caco-2, MDCK, RRCK, and others. PAMPA-based passive permeability records account for 67% (n = 3767), among which 91% of the datapoints (n = 3462) are from one publication^29^. This publication contains log P_eff_ data measured under consistent experimental conditions, making it the largest source of consistent macrocycle permeability data available in the public domain. The next highest number of datapoint records originates from the Caco-2 assay, comprising 26% (n = 1502) of the datapoints. The largest categories of entries from the Caco-2 assay have Log P_app_ AB (permeability in the apical to basolateral direction) and Log P_app_ BA (permeability in the basolateral to apical direction), together with their efflux inhibited versions Log P_app_ AB + Inh and Log P_app_ BA + Inh, determined in the presence of a cocktail of efflux inhibitors, as endpoints. The efflux ratio (ER = P_app_ AB/P_app_ BA) reveals whether a compound undergoes active efflux, i.e. if it is actively transported out of the cells and how fast this transport is compared to passive uptake into the cells, while the ER + Inh shows to what extent the transporter mediated efflux can be blocked by inhibitors. Another commonly used cell-based permeability assay, using MDCK cells, had 264 datapoints with Log P_app_ AB and ER as the two major endpoints reported. Not many macrocycles have RRCK data (n = 7), while 98 cell permeability datapoints originate from other types of assays.

**Table 1:** Number of datapoints for different permeability endpoints and types of assays.

Endpoints	PAMPA	Caco-2	MDCK	RRCK	Others	Total
Log P_eff_	3462^29,31,32,34,35^					3462
Log P_app_	305^36–58^	74^32,38,59–69^	16^51,69–73^		52^39,41,74–81^	447
Log P_app_ + Inh.		1^82^				1
Log P_app_ AB		414^7,37,46,58,82–125^	114^126–132^	2^133^		530
Log P_app_ BA		337^7,37,58,83,87,88,90,91,93–95,97–102,105–107,109–112,118,122,125^	7^37,132^	2^133^		346
Log P_app_ AB + Inh.		119^7,37,85,86,100,118,121,134^	5^37^			124
Log P_app_ BA + Inh.		104^7,37,100,118^	5^37^			109
ER		342^7,32,37,67,68,82,84,90,99,100,102,105–112,118,121,123,135^	112^37,69,72,107,126,128,129,131,132,136,137^	3^133,136^	46^35,41,76,77,138^	503
ER + Inh.		111^7,37,100,107,118,121^	5^37^			116

*Abbreviation:* PAMPA: Parallel Artificial Membrane Permeability Assay; Caco: Colorectal Adenocarcinoma Cells; MDCK: Madin-Darby Canine Kidney Cells; RRCK: Ralph Russ Canine Kidney Cells; ER: Efflux Ratio; P_app_: Apparent Permeability, P_eff_: Effective Permeability.

### Macrocycle diversity

TMAP^30^, a tree-based high-dimensional visualization tool, which provides both local and distant structural cluster information, was used to characterize the structural diversity of the macrocycles in the dataset (Fig. 3b). TMAP clearly illustrates that the permeability data provided in the web server originates from a structurally very diverse set of macrocycles. In addition, mapping of the membrane permeability assays on the TMAP tree reveals that the data from the three major assays (PAMPA, Caco-2 and MDCK) has been generated for macrocycles that show a large structural diversity. The dataset consists of both nonpeptidic and semipeptidic macrocycles. The semipeptides are situated on the right side of the TMAP tree, exhibiting a higher fraction of sp3 carbons and larger macrocyclic rings compared to the nonpeptide macrocycles, which predominantly originate from the dataset reported by Rzepiela, *et al*. (n = 3462)^29^.

### Molecular property analysis

To assess the diversity of the molecular properties of the macrocycles in the database, we analysed the distribution of key 2D molecular descriptors representing size [molecular weight (MW), macrocycle ring size (MRS)], polarity [hydrogen bond acceptors (HBA), hydrogen bond donors (HBD), topological polar surface area (TPSA)], lipophilicity (cLogP), flexibility [Kier flexibility index (Phi), number of rotatable bonds (NRotB)], and the peptide nature [amide ratio (AR)]. The molecular descriptors of a large number of the macrocycles in the dataset adhere to the cut-offs of Lipinski’s^2^ and Veber’s^25^ rules for drug-likeness (Fig. 3c). This is particularly true for polarity (HBA, HBD, TPSA) and lipophilicity (cLogP), while close to half of the macrocycles have a MW above the 500 Da cutoff. More than 350 compounds, accounting for 9% of the macrocycles in the dataset, reside in the beyond the rule of five space (bRo5) as defined by Doak, *et al*.^5^. These compounds have the potential to modulate difficult-to-drug targets, including those with extensive, flat, or groove-shaped binding sites, as well as protein-protein interactions, while still allowing for oral administration^3^.

### Quantification of peptide and nonpeptide macrocycles

**Fig. 4:**
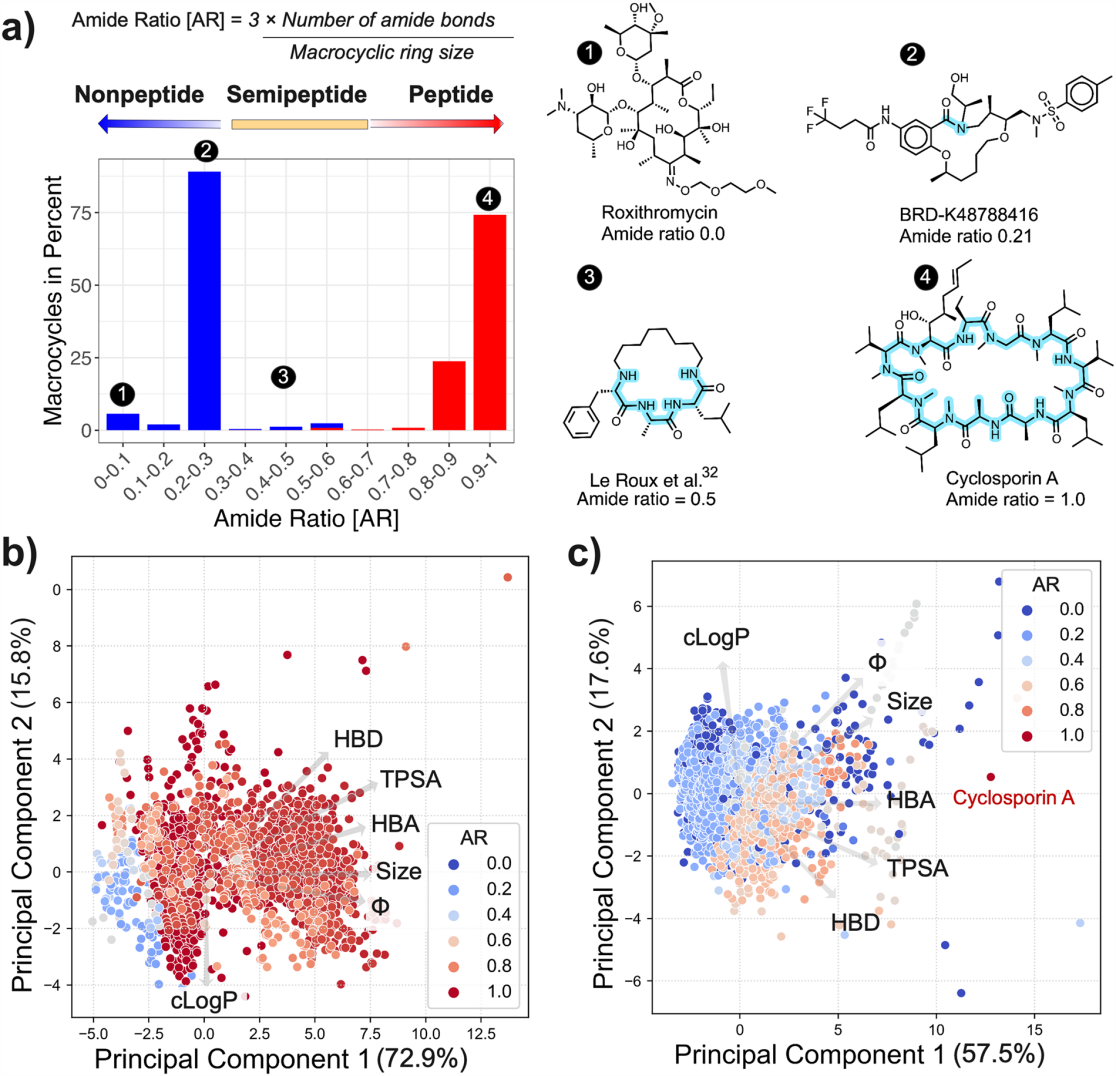
(**a**) Distribution of the amide ratio (AR) of macrocycles obtained by combination of the macrocycles from the nonpeptidic database reported herein (blue bars, n = 4216) and the cyclic peptide database (CycPeptMPDB^16^, red bars, n = 7849). The figure has been made so that the macrocycles from each database make up 100%. Classification of macrocycles by AR is shown above the figure. Representative examples of nonpeptidic (**1** and **2**), semipeptidic (**3**) and peptidic (**4**) macrocycles are shown for low to high AR values. Amide bonds within the macrocyclic ring have been shaded in blue. (**b**) Principal component analysis (PCA) comparing the chemical space of cyclic peptides (CycPeptMPDB) and nonpeptides and semipeptides from the database reported herein, with descriptor contributions highlighted by arrows. The first two principal components explain 88.7% of the variance in the dataset. (**c**) The chemical space of macrocycles reported in this study depicted using the first two principal components, which explain 75.1% of the variance in the dataset. Macrocycles are colored according to their amide ratio (AR) with blue to red circles in the two PCAs. The PCAs were constructed using the 10 descriptors provided for each macrocycle in the database.

Since no metric that quantifies whether a macrocycle is nonpeptidic, semipeptidic or peptidic has been generally accepted, we proposed the amide ratio (AR) of the macrocyclic ring as a simple descriptor for quantification of the peptidic nature of macrocycles (see Methods section). Combination of the macrocycles from the nonpeptidic database reported herein and the cyclic peptide database (CycPeptMPDB)^16^ revealed that the three classes were well differentiated by the proposed AR cut offs and also validated that the cut offs reflect the terminology used in the literature (Fig. 4a). For instance, the vast majority of the compounds in the CycPeptMPDB including the drug cyclosporin A are classified as peptidic by the AR, while semipeptides^31–33^, are also classified in agreement with the original publications.

A principal component analysis (PCA) of the combined set of the macrocycles from the nonpeptidic database reported herein and the cyclic peptide database (CycPeptMPDB)^16^ confirmed that nonpeptidic and peptidic macrocycles populated different parts of chemical space (Fig. 4b). Semipeptides were found in several regions, with most being found in between the nonpeptide and peptide classes. As expected most cyclic peptides were larger, more polar (higher TPSA and HBD count) and more flexible (higher Kier index, Φ) than the nonpeptidic macrocycles, which were somewhat more lipophilic. A separate PCA of only the nonpeptidic macrocycles from this database, but with cyclosporin A included as reference, showed a similar trend of semipeptides being more polar than the nonpeptides (Fig. 4c). Cyclosporin A was located in a chemical space far from the two other classes.

## Usage Notes

The complete dataset consisting of the 4216 unique macrocycles, their molecular descriptors and the 5638 permeability datapoints available for them can be accessed at a webserver located at https://swemacrocycledb.com/^28^. The webserver offers three primary options for accessing and handling macrocyclic cell permeability data in the *Browse, Download, and Statistic* sections.

In the *Browse* section users can select permeability datasets for macrocycles they judge to be of interest. Users can select macrocycles by unique ID, name, assay type, molecular weight, endpoints, or a combination thereof and download the data as a CSV file. Clicking on each unique molecule ID in the selected set opens a separate window displaying the name and structure, permeability data, and molecular descriptors for the selected macrocycle. Additionally, any other permeability endpoints available for the macrocycle are provided, just as a list of similar macrocycles based on the same ‘macrocyclic ring’. These functionalities of the webserver help users to find all permeability endpoints reported for a macrocycle, and directs the user to neighbouring compounds and their molecular characteristics. The *Download* section allows users to download the full dataset or subsets selected by the user as a CSV file which includes the structure, cell permeability, and molecular descriptors of the macrocycles, including their peptidic nature and the original source of the permeability data. In the *Statistics* section users can analyse both cell permeability data and molecular descriptors for the overall dataset, and the three major subsets by permeability endpoint.

## Supplementary information

Supplementary Information

## Data Availability

All the data connected to this article is available without restriction on the https://swemacrocycledb.com/ webserver. All source code is available on the GitHub (https://github.com/Macrocycle-Cell-Permeability/NPMMP-DB) and 10.6084/m9.figshare.26964259 with no restrictions to access.
