# Supplementary material for: A membrane permeability database for nonpeptidic macrocycles
Source: Sci Data. 2025 Jan 3;12:10. doi: 10.1038/s41597-024-04302-z (PMC11698989; doi:10.1038/s41597-024-04302-z)
Supplement: Supplementary file 1 — Supplementary Information [file 41597_2024_4302_MOESM1_ESM.docx]

A Membrane Permeability Database for Nonpeptidic Macrocycles

Qiushi Feng, Danjo De Chavez, Jan Kihlberg,* Vasanthanathan, Poongavanam*

**Affiliations**

Department of Chemistry-BMC, Uppsala University, SE-75123, Uppsala, Sweden

Corresponding author(s): Jan Kihlberg ([jan.kihlberg@kemi.uu.se](mailto:jan.kihlberg@kemi.uu.se)); Vasanthanathan Poongavanam ([Vasanthanathan.poongavanam@kemi.uu.se](mailto:Vasanthanathan.poongavanam@kemi.uu.se))

**Supplementary Information**

Table of Content[Supplementary Tables 2](#_Toc175313108)

[Table S1. List of Sources 2](#_Toc175313109)

[Table S2. Explanation of terminology used in the database 12](#_Toc175313110)

[Supplementary Notes 15](#_Toc175313111)

# ****Supplementary Tables****

## Table S1. List of sources for the database

| Citation | Source | Type | Link | Number of Entries |
| --- | --- | --- | --- | --- |
| Rzepiela et al., 2022 | 10.1021/acs.jmedchem.1c02090 | Journals | https://doi.org/10.1021/acs.jmedchem.1c02090 | 3348 |
| Over et al., 2016 | 10.1038/nchembio.2203 | Journals | https://doi.org/10.1038/nchembio.2203 | 849 |
| Miyachi et al., 2021 | 10.1016/j.bmcl.2021.127847 | Journals | https://doi.org/10.1016/j.bmcl.2021.127847 | 183 |
| Le Roux et al., 2020 | 10.1021/acs.jmedchem.0c00013 | Journals | https://doi.org/10.1021/acs.jmedchem.0c00013 | 122 |
| Pennington et al., 2021 | WO2021108628A1 | Patents | https://patentimages.storage.googleapis.com/ed/4b/d0/7bb0780fdf540a/WO2021108628A1.pdf | 86 |
| Hoveyda et al., 2011 | WO2011053821A1 | Patents | https://patentimages.storage.googleapis.com/f2/a4/4b/9c9ec4b8eb5fe0/WO2011053821A1.pdf | 72 |
| Nožinić et al., 2010 |  | Journals | https://hrcak.srce.hr/62210 | 65 |
| Zhang et al., 2022 | 10.1021/acs.jmedchem.2c00611 | ChEMBL | https://doi.org/10.1021/acs.jmedchem.2c00611 | 63 |
| L'Exact et al., 2023 | 10.1016/j.bbamem.2023.184196 | Journals | https://doi.org/10.1016/j.bbamem.2023.184196 | 58 |
| Liederer et al., 2006 | 10.1021/jm050277f | ChEMBL | https://doi.org/10.1021/jm050277f | 48 |
| Himmelbauer et al., 2019 | 10.1021/acs.jmedchem.9b01206 | ChEMBL | https://doi.org/10.1021/acs.jmedchem.9b01206 | 40 |
| Mackman et al., 2018 | 10.1021/acs.jmedchem.8b00802 | ChEMBL | https://doi.org/10.1021/acs.jmedchem.8b00802 | 37 |
| Lerchner et al., 2010 | 10.1016/j.bmcl.2009.11.092 | ChEMBL | https://doi.org/10.1016/j.bmcl.2009.11.092 | 36 |
| Rossi Sebastiano et al., 2018 | 10.1021/acs.jmedchem.8b00347 | Journals | https://doi.org/10.1021/acs.jmedchem.8b00347 | 36 |
| Corte et al., 2019 | 10.1021/acs.jmedchem.9b01768 | ChEMBL | https://doi.org/10.1021/acs.jmedchem.9b01768 | 31 |
| Lin et al., 2011 | 10.1124/dmd.110.034629 | ChEMBL | https://doi.org/10.1124/dmd.110.034629 | 31 |
| Marsault et al., 2008 | EP2054429B1 | Patents | https://patentimages.storage.googleapis.com/58/f4/9a/23b4a38cabb02b/EP2054429B1.pdf | 27 |
| Lücking et al., 2015 | WO2015155197A1 | Patents | https://patentimages.storage.googleapis.com/f4/9e/72/7597d53070898d/WO2015155197A1.pdf | 24 |
| Lindsley et al., 2007 | 10.1016/j.bmcl.2007.04.072 | ChEMBL | https://doi.org/10.1016/j.bmcl.2007.04.072 | 22 |
| Wang et al., 2016 | 10.1021/acs.jcim.6b00237 | Journals | https://doi.org/10.1021/acs.jcim.6b00237 | 21 |
| Ladziata et al., 2016 | 10.1016/j.bmcl.2016.08.088 | ChEMBL | https://doi.org/10.1016/j.bmcl.2016.08.088 | 16 |
| Fang et al., 2020 | 10.1016/j.bmcl.2020.126949 | ChEMBL | https://doi.org/10.1016/j.bmcl.2020.126949 | 16 |
| Moreau et al., 2014 | 10.1021/jm400121t | ChEMBL | https://doi.org/10.1021/jm400121t | 16 |
| Moore et al., 2007 | 10.1016/j.bmcl.2007.08.040 | ChEMBL | https://doi.org/10.1016/j.bmcl.2007.08.040 | 15 |
| Nilsson et al., 2010 | 10.1016/j.bmcl.2010.05.029 | ChEMBL | https://doi.org/10.1016/j.bmcl.2010.05.029 | 14 |
| Johnson et al., 2014 | 10.1021/jm500261q | ChEMBL | https://doi.org/10.1021/jm500261q | 14 |
| Zhang et al., 2016 | 10.1021/acs.jmedchem.6b00469 | ChEMBL | https://doi.org/10.1021/acs.jmedchem.6b00469 | 12 |
| Begnini et al., 2021 | 10.1021/acsmedchemlett.1c00126 | Journals | https://doi.org/10.1021/acsmedchemlett.1c00126 | 12 |
| Hoveyda et al., 2008 | WO2008130464A1 | Patents | https://patentimages.storage.googleapis.com/2a/a0/d3/ac820476e213f6/WO2008130464A1.pdf | 12 |
| Lücking et al., 2017 | WO2017055196A1 | Patents | https://patentimages.storage.googleapis.com/29/84/ee/a48d88e05771a4/WO2017055196A1.pdf | 12 |
| Yamaguchi-Sasaki et al., 2020 | 10.1016/j.bmc.2020.115818 | ChEMBL | https://doi.org/10.1016/j.bmc.2020.115818 | 10 |
| Corte et al., 2017 | 10.1016/j.bmcl.2017.06.058 | ChEMBL | https://doi.org/10.1016/j.bmcl.2017.06.058 | 10 |
| Liu et al., 2021 | 10.1021/acs.jmedchem.1c00712 | ChEMBL | https://doi.org/10.1021/acs.jmedchem.1c00712 | 10 |
| Sund et al., 2011 | 10.1016/j.bmcl.2010.10.140 | ChEMBL | https://doi.org/10.1016/j.bmcl.2010.10.140 | 9 |
| Begnini et al., 2022 | 10.1021/acs.jmedchem.1c01975 | ChEMBL | https://doi.org/10.1021/acs.jmedchem.1c01975 | 9 |
| Wang et al., 2017 | 10.1021/acs.jmedchem.7b00017 | ChEMBL | https://doi.org/10.1021/acs.jmedchem.7b00017 | 9 |
| Ahlbach et al., 2015 | 10.4155/fmc.15.78 | Journals | https://doi.org/10.4155/fmc.15.78 | 9 |
| Saunders et al., 2022 | 10.1002/anie.202206866 | Journals | https://doi.org/10.1002/anie.202206866 | 8 |
| Raboisson et al., 2008 | 10.1016/j.bmcl.2008.07.088 | ChEMBL | https://doi.org/10.1016/j.bmcl.2008.07.088 | 8 |
| Andersson et al., 2016 | 10.1021/acs.jmedchem.5b01871 | Journals | https://doi.org/10.1021/acs.jmedchem.5b01871 | 8 |
| DeGoey et al., 2017 | 10.1021/acs.jmedchem.7b00717 | ChEMBL | https://doi.org/10.1021/acs.jmedchem.7b00717 | 8 |
| Richter et al., 2017 | 10.1021/acsmedchemlett.6b00375 | ChEMBL | https://doi.org/10.1021/acsmedchemlett.6b00375 | 8 |
| Vendeville et al., 2008 | 10.1016/j.bmcl.2008.10.004 | ChEMBL | https://doi.org/10.1016/j.bmcl.2008.10.004 | 7 |
| Kaneda et al., 2018 | 10.1021/acsmedchemlett.8b00028 | ChEMBL | https://doi.org/10.1021/acsmedchemlett.8b00028 | 7 |
| Clark et al., 2019 | 10.1016/j.bmcl.2019.08.008 | ChEMBL | https://doi.org/10.1016/j.bmcl.2019.08.008 | 6 |
| Basit et al., 2017 | 10.1016/j.ejmech.2017.04.032 | ChEMBL | https://doi.org/10.1016/j.ejmech.2017.04.032 | 6 |
| Li et al., 2018 | 10.1021/acs.jmedchem.8b00819 | ChEMBL | https://doi.org/10.1021/acs.jmedchem.8b00819 | 6 |
| Tyagi et al., 2018 | 10.1021/acs.orglett.8b02447 | Journals | https://doi.org/10.1021/acs.orglett.8b02447 | 6 |
| William et al., 2011 | 10.1021/jm200326p | ChEMBL | https://doi.org/10.1021/jm200326p | 6 |
| Hoveyda et al., 2011 | 10.1021/jm2007062 | ChEMBL | https://doi.org/10.1021/jm2007062 | 6 |
| ChEMBL | ChEMBL | ChEMBL | Not Available | 6 |
| Yang et al., 2023 | EP4074715A1 | Patents | https://patentimages.storage.googleapis.com/d6/53/bc/317137008ae45e/EP4074715A1.pdf | 6 |
| Kettle et al., 2016 | 10.1021/acs.jmedchem.5b01760 | ChEMBL | https://doi.org/10.1021/acs.jmedchem.5b01760 | 5 |
| Giroud et al., 2018 | 10.1021/acs.jmedchem.7b01869 | ChEMBL | https://doi.org/10.1021/acs.jmedchem.7b01869 | 5 |
| Wieske et al., 2023 | 10.1002/chem.202202798 | Journals | https://doi.org/10.1002/chem.202202798 | 4 |
| Kostrun et al., 2021 | 10.1021/acs.jmedchem.1c00327 | ChEMBL | https://doi.org/10.1021/acs.jmedchem.1c00327 | 4 |
| Naylor et al., 2018 | 10.1021/acs.jmedchem.8b01259 | ChEMBL | https://doi.org/10.1021/acs.jmedchem.8b01259 | 4 |
| Jiang et al., 2014 | 10.1021/jm400164c | ChEMBL | https://doi.org/10.1021/jm400164c | 4 |
| Rosenquist et al., 2014 | 10.1021/jm401507s | ChEMBL | https://doi.org/10.1021/jm401507s | 4 |
| De Kock et al., 2007 | WO2007014918A1 | Patents | https://patentimages.storage.googleapis.com/0c/93/f1/dadf2867b81398/WO2007014918A1.pdf | 4 |
| Jing et al., 2014 | 10.1016/j.bmc.2013.11.037 | ChEMBL | https://doi.org/10.1016/j.bmc.2013.11.037 | 3 |
| Boy et al., 2015 | 10.1016/j.bmcl.2015.10.031 | ChEMBL | https://doi.org/10.1016/j.bmcl.2015.10.031 | 3 |
| Wurtz et al., 2017 | 10.1016/j.bmcl.2017.04.008 | ChEMBL | https://doi.org/10.1016/j.bmcl.2017.04.008 | 3 |
| Zhang et al., 2021 | 10.1021/acs.jmedchem.1c00296 | ChEMBL | https://doi.org/10.1021/acs.jmedchem.1c00296 | 3 |
| Cee et al., 2016 | 10.1021/acsmedchemlett.5b00403 | ChEMBL | https://doi.org/10.1021/acsmedchemlett.5b00403 | 3 |
| Li et al., 2017 | 10.1021/acsmedchemlett.6b00384 | ChEMBL | https://doi.org/10.1021/acsmedchemlett.6b00384 | 3 |
| Stachel et al., 2006 | 10.1021/jm060884i | ChEMBL | https://doi.org/10.1021/jm060884i | 3 |
| William et al., 2012 | 10.1021/jm201112g | ChEMBL | https://doi.org/10.1021/jm201112g | 3 |
| Joshi et al., 2013 | 10.1021/jm400811d | ChEMBL | https://doi.org/10.1021/jm400811d | 3 |
| Peng et al., 2010 | 10.1021/jm900672t | ChEMBL | https://doi.org/10.1021/jm900672t | 3 |
| Halland et al., 2014 | 10.1021/ml4004556 | ChEMBL | https://doi.org/10.1021/ml4004556 | 3 |
| Gozalbes et al., 2011 | 10.1016/j.bmc.2011.03.011 | ChEMBL | https://doi.org/10.1016/j.bmc.2011.03.011 | 2 |
| Lampa et al., 2014 | 10.1016/j.bmc.2014.10.010 | ChEMBL | https://doi.org/10.1016/j.bmc.2014.10.010 | 2 |
| Machauer et al., 2009 | 10.1016/j.bmcl.2009.01.055 | ChEMBL | https://doi.org/10.1016/j.bmcl.2009.01.055 | 2 |
| McGowan et al., 2012 | 10.1016/j.bmcl.2012.03.097 | ChEMBL | https://doi.org/10.1016/j.bmcl.2012.03.097 | 2 |
| Maccari et al., 2014 | 10.1016/j.bmcl.2014.09.081 | ChEMBL | https://doi.org/10.1016/j.bmcl.2014.09.081 | 2 |
| Parsy et al., 2015 | 10.1016/j.bmcl.2015.09.009 | ChEMBL | https://doi.org/10.1016/j.bmcl.2015.09.009 | 2 |
| Granger & Brown, 2016 | 10.1016/j.bmcl.2016.09.039 | ChEMBL | https://doi.org/10.1016/j.bmcl.2016.09.039 | 2 |
| Farand et al., 2016 | 10.1016/j.bmcl.2016.10.092 | ChEMBL | https://doi.org/10.1016/j.bmcl.2016.10.092 | 2 |
| Xiao et al., 2021 | 10.1016/j.bmcl.2021.128354 | ChEMBL | https://doi.org/10.1016/j.bmcl.2021.128354 | 2 |
| Yang et al., 2020 | 10.1021/acs.jmedchem.0c00464 | ChEMBL | https://doi.org/10.1021/acs.jmedchem.0c00464 | 2 |
| Randolph et al., 2020 | 10.1021/acs.jmedchem.0c00956 | ChEMBL | https://doi.org/10.1021/acs.jmedchem.0c00956 | 2 |
| Begnini et al., 2020 | 10.1021/acs.jmedchem.0c01569 | Journals | https://doi.org/10.1021/acs.jmedchem.0c01569 | 2 |
| Wuelfing et al., 2022 | 10.1021/acs.jmedchem.1c01687 | ChEMBL | https://doi.org/10.1021/acs.jmedchem.1c01687 | 2 |
| Wang et al., 2022 | 10.1021/acs.jmedchem.2c00981 | ChEMBL | https://doi.org/10.1021/acs.jmedchem.2c00981 | 2 |
| Corte et al., 2017 | 10.1021/acs.jmedchem.6b01460 | ChEMBL | https://doi.org/10.1021/acs.jmedchem.6b01460 | 2 |
| McCoull et al., 2017 | 10.1021/acs.jmedchem.7b00359 | ChEMBL | https://doi.org/10.1021/acs.jmedchem.7b00359 | 2 |
| Giroud, Kuhn, et al., 2018 | 10.1021/acs.jmedchem.7b01870 | ChEMBL | https://doi.org/10.1021/acs.jmedchem.7b01870 | 2 |
| Do et al., 2019 | 10.1021/acs.jmedchem.8b02032 | ChEMBL | https://doi.org/10.1021/acs.jmedchem.8b02032 | 2 |
| Adebesin et al., 2019 | 10.1021/acs.jmedchem.9b00952 | ChEMBL | https://doi.org/10.1021/acs.jmedchem.9b00952 | 2 |
| Engelhardt et al., 2019 | 10.1021/acs.jmedchem.9b01169 | ChEMBL | https://doi.org/10.1021/acs.jmedchem.9b01169 | 2 |
| Rescourio et al., 2019 | 10.1021/acs.jmedchem.9b01310 | ChEMBL | https://doi.org/10.1021/acs.jmedchem.9b01310 | 2 |
| Spencer et al., 2020 | 10.1021/acsmedchemlett.0c00061 | Journals | https://doi.org/10.1021/acsmedchemlett.0c00061 | 2 |
| Xin et al., 2020 | 10.1021/acsmedchemlett.9b00611 | ChEMBL | https://doi.org/10.1021/acsmedchemlett.9b00611 | 2 |
| William et al., 2012 | 10.1021/jm201454n | ChEMBL | https://doi.org/10.1021/jm201454n | 2 |
| Parsy et al., 2010 | WO2010118078A1 | Patents | https://patentimages.storage.googleapis.com/d6/e3/ea/f18f11e25984a8/WO2010118078A1.pdf | 2 |
| Du-Cuny et al., 2009 | 10.1016/j.bmc.2009.08.022 | ChEMBL | https://doi.org/10.1016/j.bmc.2009.08.022 | 1 |
| Raboisson, Lin, et al., 2008 | 10.1016/j.bmcl.2008.07.124 | ChEMBL | https://doi.org/10.1016/j.bmcl.2008.07.124 | 1 |
| Duan et al., 2012 | 10.1016/j.bmcl.2012.02.039 | ChEMBL | https://doi.org/10.1016/j.bmcl.2012.02.039 | 1 |
| Xue et al., 2020 | 10.1016/j.ejmech.2020.112465 | ChEMBL | https://doi.org/10.1016/j.ejmech.2020.112465 | 1 |
| Tess et al., 2020 | 10.1021/acs.jmedchem.0c01033 | ChEMBL | https://doi.org/10.1021/acs.jmedchem.0c01033 | 1 |
| Zheng et al., 2021 | 10.1021/acs.jmedchem.1c00607 | ChEMBL | https://doi.org/10.1021/acs.jmedchem.1c00607 | 1 |
| Dilger et al., 2021 | 10.1021/acs.jmedchem.1c00613 | ChEMBL | https://doi.org/10.1021/acs.jmedchem.1c00613 | 1 |
| Pasero et al., 2018 | 10.1021/acs.jmedchem.8b00619 | ChEMBL | https://doi.org/10.1021/acs.jmedchem.8b00619 | 1 |
| Houštecká et al., 2020 | 10.1021/acs.jmedchem.9b01351 | ChEMBL | https://doi.org/10.1021/acs.jmedchem.9b01351 | 1 |
| Stefanucci et al., 2019 | 10.1021/acs.jmedchem.9b01963 | ChEMBL | https://doi.org/10.1021/acs.jmedchem.9b01963 | 1 |
| Hostalkova et al., 2019 | 10.1021/acs.jnatprod.8b00592 | ChEMBL | https://doi.org/10.1021/acs.jnatprod.8b00592 | 1 |
| Hopkins et al., 2019 | 10.1021/acsmedchemlett.9b00036 | ChEMBL | https://doi.org/10.1021/acsmedchemlett.9b00036 | 1 |
| Hess et al., 2007 | 10.1021/jm070836d | ChEMBL | https://doi.org/10.1021/jm070836d | 1 |
| Giordanetto & Kihlberg, 2014 | 10.1021/jm400887j | ChEMBL | https://doi.org/10.1021/jm400887j | 1 |
| Xu et al., 2017 | 10.1039/C6MD00633G | ChEMBL | https://doi.org/10.1039/C6MD00633G | 1 |

## Table S2. Explanation of terminology used in the CSV files that can be downloaded from the database

| Terminology | Explanation |
| --- | --- |
| ID | Unique molecule ID provided for each macrocycle |
| Original_ID | Molecular ID/name from the original source of the data |
| Common_name | Common name of the compound |
| SMILES | SMILES (Simplified molecular-input line-entry system), in original source |
| Standardized_SMILES | SMILES standardized by RDKit |
| Value | Permeability value in the original source |
| Unit | Permeability unit of the data in original sources |
| Endpoint | Permeability endpoint of the data in the original source |
| Standardized_Value | Permeability value of the data after value standardization. *Original permeability values were converted into the unit cm/s and then their logarithmic values were calculated.* |
| Standardized_Endpoint | Permeability endpoint of the data after endpoint standardization. *The permeability data endpoints were standardised to a logarithmic format, regardless of their original presentation in the source literature as logarithmic, negative logarithmic, or non-logarithmic values.* |
| Assay | *In vitro* assay employed for permeability measurement |
| Description | Description of the permeability data in original sources |
| Link | Website link to the original source |
| Source Type | Type of original source |
| Source | DOI or patent number of the original source |
| Citation | Citation of the original source |
| Publish_Year | Publication year of the original source |
| Macrocycle_Ring_Size | Number of atoms in the main macrocycle ring |
| Macrocycle_Free_Amide_Count | Number of secondary amide bonds (CONH groups in the ring) |
| Macrocycle_Substituted_Amide_Count | Number of N-alkylated amide bonds |
| Macrocycle_Overall_Amide_Count | Number of total amide bonds |
| Macrocycle_Ring_smiles | SMILES of macrocycle ring itself |
| Macrocycle_Peripheral_smiles | SMILES of a peripheral group (a group attached to the MC ring that contains a single heavy atom) |
| Free_Amide_Ratio | $\text{Free Amide Ratio=} \frac{\text{3 × Number of Free Amide Bonds}}{\text{Macrocycle Ring Size}}$  Free Amide Ratio is based on the number of free amide bonds (NH ones) within the macrocyclic ring, multiplied by three to account for the number atoms (-C-N-C_α_-) forming each amide bond, division by the macrocycle ring size (MRS). |
| Amide_Ratio | $\text{Amide Ratio=} \frac{\text{3 × Number of Amide Bonds}}{\text{Macrocycle Ring Size}}$  Amide Ratio is based on the number of amide bonds (nAB), including both NH and N-alkylated ones, within the macrocyclic ring. |
| Num_Rings | Number of rings in the macrocycle |
| Num_Aromatic_Rings | Number of aromatic rings in the macrocycle |
| cLogP | Calculated logarithm of the octanol-water partition coefficient |
| Molecular_Weight | Molecular weight |
| Num_H_Acceptors | Number of hydrogen bond acceptors |
| Num_H_Donors | Number of hydrogen bond donors |
| Num_Heavy_Atoms | Number of heavy atoms (any atom that is not hydrogen) in the macrocycle. |
| Num_Carbon_Atoms | Number of carbon atoms in the macrocycle |
| Fraction_SP3_Carbons | Fraction of carbons that are sp3 hybridized |
| TPSA | Topological polar surface area |
| Num_Rotatable_Bonds | Number of rotatable bonds |
| Kier_index | Kier flexibility index |
| InchiKey | InChiKey of the macrocyclic compound. The 27-character InChiKey is a hashed version of the full InChI (International Chemical Identifier). |
